# Circular RNAs: emerging cancer biomarkers and targets

**DOI:** 10.1186/s13046-017-0624-z

**Published:** 2017-11-02

**Authors:** Yu Zhang, Wei Liang, Peng Zhang, Jingyan Chen, Hui Qian, Xu Zhang, Wenrong Xu

**Affiliations:** 1 Jiangsu Key Laboratory of Medical Science and Laboratory Medicine, School of Medicine, Jiangsu University, 301 Xuefu Road, Zhenjiang, Jiangsu 212013 China; 2 Institute of Digestive Diseases, The Affiliated People’s Hospital of Jiangsu University, Zhenjiang, Jiangsu 212002 China

**Keywords:** CircRNA, Cancer, ceRNA, Biomarker, Target

## Abstract

CircRNAs are a class of RNA molecules that structurally form closed loops. CircRNAs are abundant in eukaryotic transcripts and show certain levels of tissue and cell specificity. CircRNAs have been suggested to regulate gene expression at transcriptional, post-transcriptional, and translational levels. An increasing number of studies have shown that circRNAs play important roles in the development and progression of diseases including cancer. In particular, circRNAs have shown great potential in cancer diagnosis, prognosis, and therapy. In this review, we provide an overview of the biogenesis and characteristics of circRNAs, succinctly describe their functions, and comprehensively discuss about the recent advances in the roles of circRNAs in cancer with an emphasis on their clinical values.

**Electronic supplementary material:**

The online version of this article (10.1186/s13046-017-0624-z) contains supplementary material, which is available to authorized users.

## Background

Circular RNAs (circRNAs) are a class of RNA molecules that lack 5′-3′ ends and poly A tail and covalently form closed loops. Owing to this structure, circRNAs are not easily degraded by exonuclease RNase R and exist stably in the cells [1]. CircRNAs were firstly identified in viruses in 1970s and later in eukaryotic cells [2, 3]. CircRNAs were initially considered as the products of abnormal RNA splicing; therefore, they have not garnered much scientific attention. In the past few decades, technical constraints have limited the progress in circRNA research. However, following the rapid development in bioinformatics and high-throughput sequencing, the ancient and conserved characteristics of circRNAs are gradually being unveiled. Increasing evidence suggest that circRNAs are involved in the pathogenesis of a variety of diseases, including osteoarthritis, diabetes, heart failure, Alzheimer’s disease, and cancer [4–8]. In particular, circRNAs are reported to play important roles in cancer growth, metastasis, and therapy resistance [9]. Moreover, the stability of circRNAs in body fluids and the specificity of circRNAs in diseases have made them new molecular markers for cancer diagnosis [9–12].

## Biogenesis of circRNAs

**Fig. 1:**
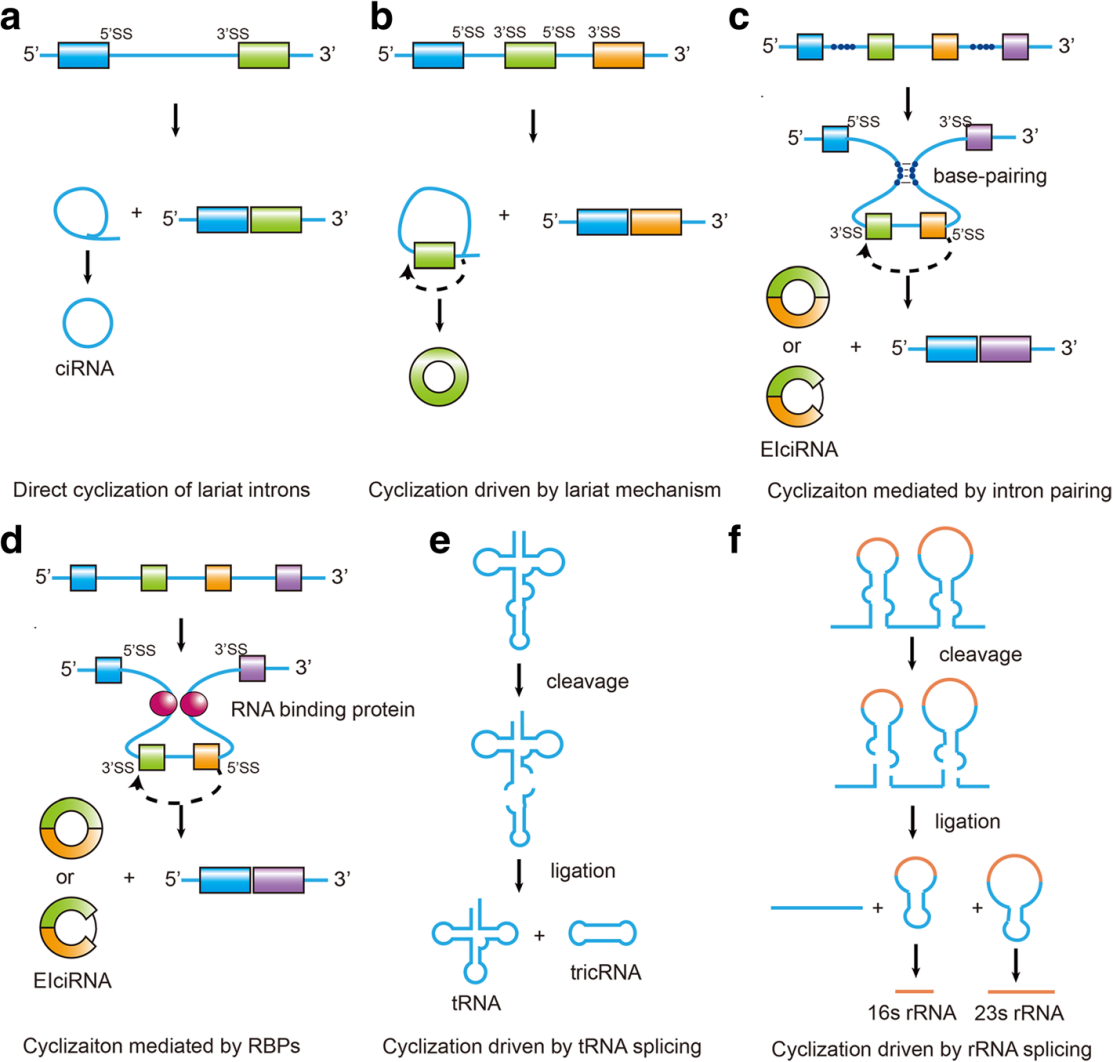
The proposed models of circRNA formation. **a** Direct cyclization of lariat introns. Canonical linear splicing generates a lariat structure. The 3′ downstream of the lariat intron is trimmed to form a circular intronic RNA (ciRNA). **b** Cyclization driven by lariat mechanism. The exon-skipping event during alternative splicing promotes the 3′ splice site (3′SS) of the exon to covalently splice to the 5′ splice site (5′SS). **c** Cyclization mediated by intron pairing. Intron pairing brings the appropriate splice signals within proximity of each other, which promotes cyclization. **d** Cyclization mediated by RNA-binding proteins (RBPs). RBPs bring the appropriate splice signals within proximity of each other, which promotes cyclization. **e** Cyclization driven by tRNA splicing. **f** Cyclization driven by rRNA splicing

**Fig. 2:**
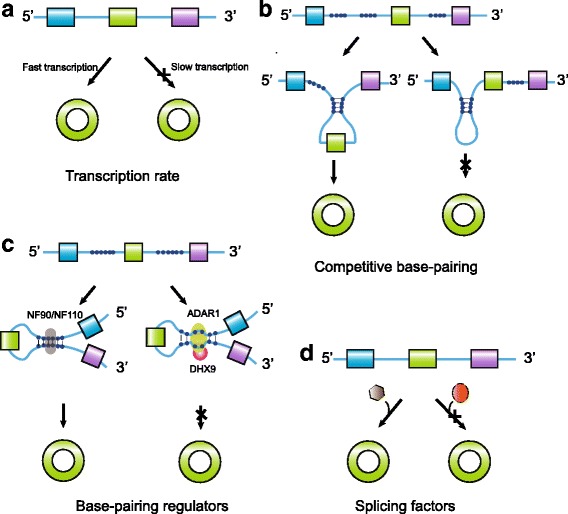
The regulation of circRNA biogenesis. **a** Competitive base-pairing. Inter**-**intronic base-pairing promotes the biogenesis of circRNAs, while intra**-**intronic base-pairing inhibits the biogenesis of circRNAs. **b** Base-pairing regulators. **c**. Splicing factors have dual roles in the biogenesis of circRNAs. **d** The transcription rate of circRNA producing gene influences the expression of circRNA

**Fig. 3:**
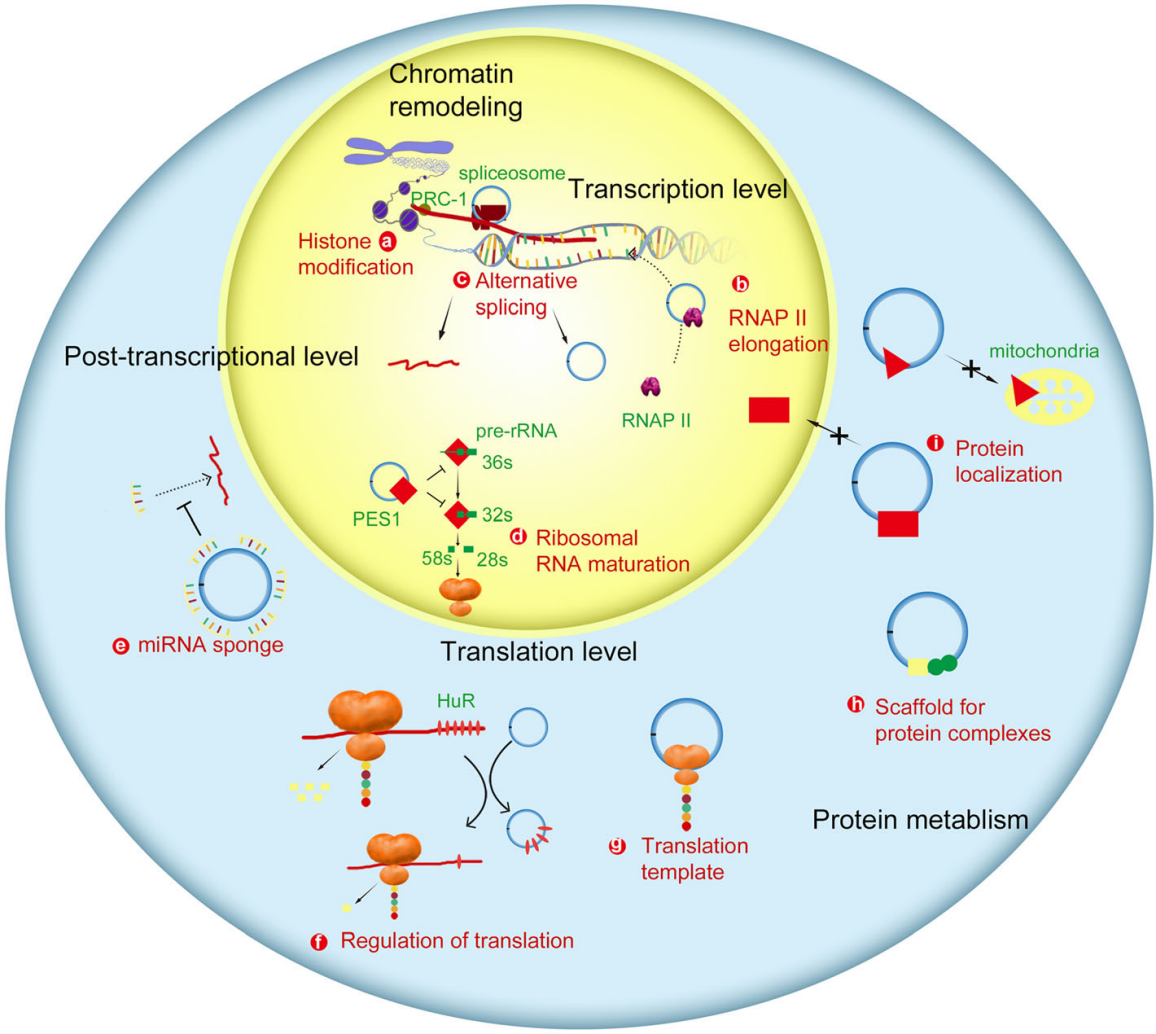
The functions of circular RNAs. CircRNAs can impact genetic output at almost every stage of a gene’s life cycle—from epigenetic regulation to transcriptional and posttranscriptional control to translational control. Listed are some gene regulation functions of circRNAs

**Fig. 4:**
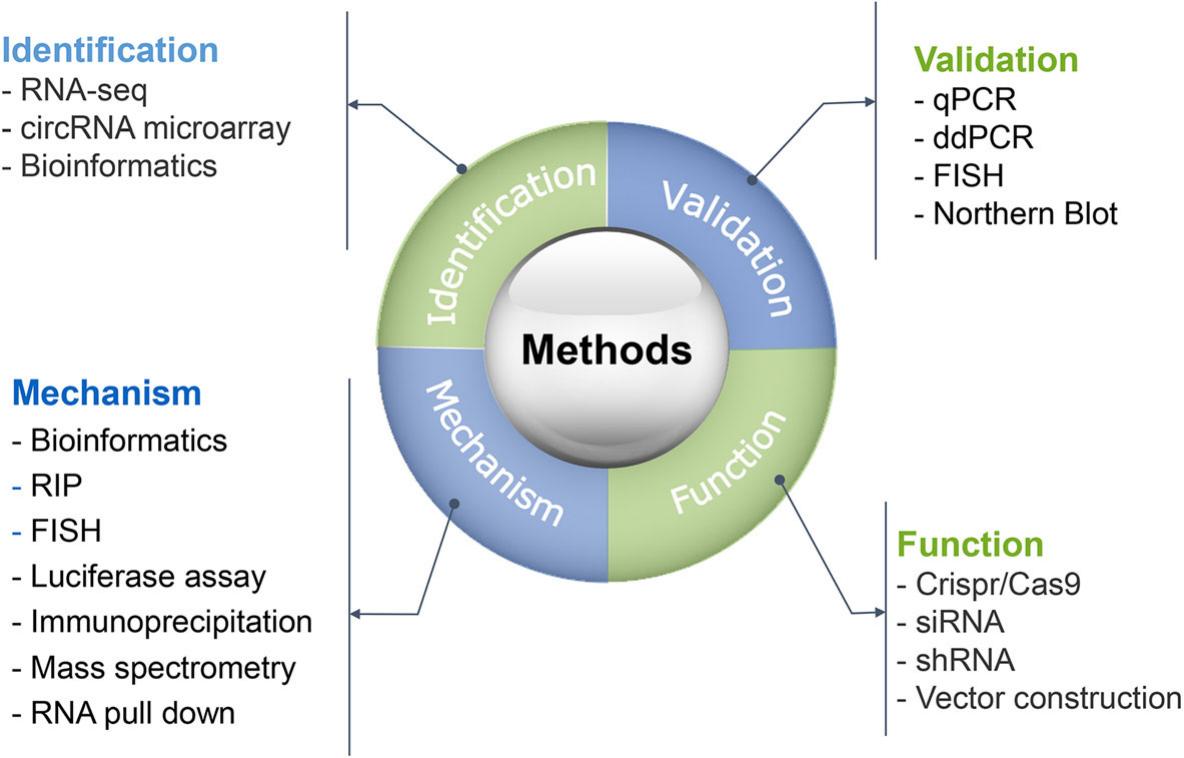
The strategies for circRNA research .

In contrast to linear RNAs that are formed by classical splicing, circRNAs are formed by back-splicing [13]. Nascent circRNAs are generally identified later than linear RNAs, suggesting that most circRNAs are produced after transcription from the parental genes [14]. Currently, 6 models have been proposed for the formation of circRNAs: (1) direct cyclization of lariat introns [13, 15] (Fig. 1a); (2) cyclization driven by lariat mechanism [13] (Fig. 1b); (3) cyclization mediated by intron pairing [13, 16] (Fig. 1c); (4) cyclization mediated by RNA-binding proteins (RBPs) and *trans*-acting factors [17] (Fig. 1d); (5) cyclization driven by tRNA splicing [18] (Fig. 1e); and (6) cyclization driven by rRNA splicing [19, 20] (Fig. 1f). In addition, all the four types of alternative splicing (namely cassette exon, intron retention, alternative donor site and alternative acceptor site) that have been identified in linear mRNA are found in circRNAs, which adds more complexity to the biogenesis of circRNAs [21].

The formation process of circRNA is precisely and tightly controlled. The process of circRNA formation is influenced by the transcription rate of the corresponding gene. The transcription rate of circRNA-producing genes is significantly higher than that of non-circRNA-producing genes (Fig. 2a). The steady-state levels of circRNAs are positively correlated with their nascent levels [14]. The cis-acting element inside RNA (reverse complementary sequence or RBP binding sequence) can promote the formation of circRNAs. The competitive base-pairing between different pairs of complementary regions may influence backsplicing efficiency (Fig. 2b) [22]. Some proteins can bind to and stabilize the complementary sequence, while others may cleave the complementary sequence [23–25]. For example, DExH-box helicase 9 (DHX9), an abundant nuclear RNA helicase, interacts specifically with adenosine deaminase acting on RNA-1 (ADAR) and reduces the formation of circRNA by recognizing and unpackaging the RNA double-stranded structure formed by the reverse complementary element (Fig. 2c) [23, 24]. On the contrary, NF90/NF110 protein promotes circRNA production in the nucleus by binding to and stabilizing complementary sequences (Fig. 2c) [25]. Additionally, various splicing factors, such as fused in sarcoma (FUS), SR protein, heterogeneous nuclear ribonucleoprotein (hnRNP), and Quaking (QKI), have been found to regulate the formation of circRNAs (Fig. 2d) [26–29]. The effect of these RNA-binding proteins on circRNA biogenesis is very complex, involving both positive and negative regulations. For example, FUS and hnRNPL positively affect the biogenesis of some circRNAs and negatively affect that of other circRNAs [26, 29]. Moreover, the biogenesis of some circRNAs is specifically controlled by certain splicing factors [30].

## Characteristics of circRNAs

CircRNAs are a class of stable RNA molecules that are evolutionarily conserved in mammalian cells. More than a million circRNAs exist in human tissues as detected by high-throughput sequencing [31]. CircRNAs are predominantly found in the cytoplasm, whereas a small number of circRNAs are located in the nucleus. The evolution of circRNAs in different species appears to be relatively conserved [32]. Although the overall abundance of circRNAs is low, the expression of some circRNAs is much higher than that of linear RNAs [13]. The expression level and function of circRNAs are independent of linear RNA isoforms [33]. Thus, circRNA expression may contain disease-relevant information that cannot be assessed by canonical RNA analysis. Although the efficiency of circRNA formation is very low [14], the half-life of circRNA is long [34] due to its resistance to RNA exonucleases, enabling circRNAs to maintain stable levels in the body under normal conditions. However, circular RNA can be cleaved by endonucleases; therefore, RNA interference can be used to knock down circRNA expression.

## Functions of circRNAs

RNAs have great structural complexity and plasticity and can interact with both DNA and other RNAs. Regulatory RNAs are proposed to function as modular scaffolds to assemble diverse combinations of regulatory proteins, thus enhancing protein-protein interactions [35]. Regulatory RNAs can establish important biological networks through RNA-DNA, RNA-RNA, and RNA-protein interactions. Although the function of circRNAs is not entirely clear, the recent studies have shown that circRNAs may have the ability to regulate gene expression through multiple mechanisms (Table 1).

**Table 1:** Functions of circRNAs

Function	Figure 3	Example	Reference
Histone modification	a	cANRIL	[39, 40]
RNAP II elongation	b	ci-ankrd52	[15]
		EIciEIF3j	[16]
Alternative splicing	c	circMbl	[41]
RNA maturation	d	circANRIL	[42]
miRNA sponge	e	circHIPK3	[33]
		ciRS-7	[43, 44]
Translation regulation	f	circPABPN1	[45]
Translation	g	circ-ZNF609	[50]
		circMbl	[51]
Scaffold for proteins	h	circ-Foxo3	[56]
Protein localization	i	circ-Foxo3	[57]

RNAs can bind to protein complexes of the trithorax chromatin-activating or polycomb group (PcG) chromatin-repressing families and guide them to their sites of action. These complexes act antagonistically to activate or inhibit histone modifications on specific loci, which represents a global mechanism for epigenetic modification [36–38]. A previous study showed that lncRNA ANRIL (antisense non-coding RNA in the *INK4* locus) could promote PcG-mediated repression of the *INK4/ARF* locus [39]. A recent study suggests that the expression of both circular and linear *ANRIL* transcripts correlates with that of coding *INK4/ARF* transcripts and the relevance is even stronger for circular ANRIL (cANRIL) [40]. cANRIL may regulate INK4/ARF expression by competitive splicing (Fig. 3a).

CircRNAs can promote the process of gene transcription (Fig. 3b). CircRNA ci-ankrd52, which is derived from the second intron region of the *ANKRD52* gene, is located in the nucleus. The RNA terminal sequence of ci-ankrd52 has a typical characteristic element, which can prevent the decomposition of lariat debranching enzyme to ensure its circular structure. CircRNA ci-ankrd52 accumulates to its sites of transcription to promote *ANKRD52* gene transcription, associates with elongation Pol II machinery, and acts as a positive regulator of Pol II transcription [15]. Another study demonstrates that circRNA EIciEIF3j could promote the transcription of its parent gene *EIF3J* by combining with snRNPs (small nuclear ribonucleoproteins) and Pol II [16].

CircRNAs are involved in the regulation of selective splicing (Fig. 3c). Ashwal-Fluss et al. demonstrate that circMbl stems from the circularization of the second exon of the muscleblind gene (*MBL*) and circMbL could compete with linear *MBL* mRNA for selective splicing [41]. Notably, MBL could interact with circMbl and its flanking introns and promote exon circulation. In addtion, when the efficiency of linear splicing increases, circRNA abundance decreases, which suggests that there is a genome-wide competition between canonical splicing and circRNA generation.

CircRNAs can inhibit the maturation of RNA (Fig. 3d). Holdt et al. demonstrat that circANRIL binds to the C-terminal lysine-rich domain of PES1 to prevent pre-rRNA binding and exonuclease-mediated rRNA maturation, further affecting ribosome formation and the translation processes [42].

CircRNAs can function as miRNA sponges (Fig. 3e) [43, 44]. The regulation of RNA activity can be achieved via competitive binding at a common microRNA response element (MRE). Some circRNAs harbor MREs and can serve as miRNA sponges in the cell by binding to a miRNA, which removes the inhibitory effect of miRNA on its target genes and further up-regulates the expression of the target genes. For example, circHIPK3 can promote the growth of human cancer cells by competitively binding to the tumor-suppressive miR-124 [33]. Additionally, ciRS-7 contains more than 70 selectively conserved miRNA binding sites, and is a miRNA antagonist with the miRNA-binding capacity 10 times higher than that of any other known transcripts [43, 44].

CircRNAs are involved in the translation process (Fig. 3f). A recent study suggests that circRNAs can regulate the translation process by competing with mRNA for binding to RBP. For example, circPABPN1 binds to HuR, which hinders the binding of *PABPN1* mRNA to HuR, thus impairing the translation process of PABPN1 [45]. Additionally, several studies have shown that after inserting an internal ribosome entry site (IRES) into a synthetic circRNA, the eukaryotic ribosomal 40S subunit would bind to circRNAs at the IRES and initiate the translation process [46–48]. The previous studies have shown that circRNAs in hepatitis D virus (HDV) could encode the hepatitis D virus antigen (HDAg) after infecting eukaryotic cells [49]. The recent studies suggest that endogenous eukaryotic circRNAs could also encode proteins (Fig. 3g) [50–52]. Circ-ZNF609, which contains an open reading frame spanning from the start codon in common with the linear transcript, is able to translate a protein during myogenesis in human and murine cells, but the protein translation efficiency is lower than that of linear RNA [50]. The protein translation activity of circ-ZNF609 is driven by IRES. In drosophila, a circRNA generated from the *muscleblind* locus encodes a 37.04 kDa protein, which is confirmed by mass spectrometry analysis [51]. These circRNAs have no free 5′ and 3′ ends and are translated in a 5′-hat-independent manner. IRES and m6A modifications have been reported to be related to the translation process of circRNAs. CircRNAs recruit YTHDF3 through the m6A modification site and then recruit EIf4G2, which in turn initiates the protein translation process [52]. In eukaryotic cells, mRNA modifications of m6A, Ψ, and m5C can alter the efficiency and fidelity of translation [53]. Whether other transcription-related modifications of circRNAs exist still needs further study.

CircRNAs can promote protein-protein interactions (Fig. 3h). At the G1 phase of cell cycle, CDK2 forms a complex with cyclin E. The cyclin complex phosphorylates retinoblastoma protein (Rb) and promotes gene expression, leading to the progression of cells from G1 to S phase [54]. p21 can bind CDK2 and inhibit CDK2 activity [55]. Circ-Foxo3 could interact with both p21 and CDK2 to inhibit the interaction between CDK2 and cyclin E, resulting in the inhibition of cell cycle progression [56]. CircRNA can also affect protein localization (Fig. 3i). For instance, circ-Foxo3 is co-localized with ID1 and E2F1 in the cytoplasm and could decrease nuclear translocation of ID1 and E2F1. In addition, circ-Foxo3 could decrease the distribution of HIF1α in the nucleus and that of FAK in the mitochondria in H_2_O_2_-treated cells [57].

## CircRNAs as cancer biomarkers

The expression patterns and characteristics of circRNAs (universality, conservatism, tissue/cell specificity, and stability) make them ideal candidates as biomarkers [31–34]. The detection of circRNAs in human blood, saliva, and gastric fluid also increases the potential of circRNAs as disease biomarkers [10, 11, 58, 59]. Memczak et al. observed a relatively higher level of circRNA than that of linear RNA in the blood [10]. Additionally, these circRNAs could be reproducibly and easily detected in blood samples. Overall, many circRNAs express at high levels in the blood, while the corresponding linear RNAs show average or low abundances. Thus, blood circRNA may provide disease-relevant information that cannot be revealed by canonical RNA analysis. CircRNAs have been shown to be enriched by at least 2-fold in exosomes compared to the producing cells [60]. Bahn and colleagues have identified 422 circRNAs in human cell-free saliva by bioinformatics analysis and have shown that these salivary circRNAs are involved in intercellular signalling and inflammatory responses [11]. Furthermore, circRNAs can be detected in human gastric juice. Shao et al. demonstrate that freeze-thaw for 8 cycles or storage at 4 °C for 8 h do not affect the expression levels of hsa_circ_0014717 in gastric juice [59]. Recently, many studies have explored the clinical values of circRNAs in cancer and have demonstrated that some circRNAs are not only superior to the corresponding mRNA in terms of stability and diagnostic value, but also reflect the stage characteristics of tumorigenesis, which has great potential in the diagnosis of cancers [61–64]. The recent studies on the roles and clinical significances of circRNAs in cancer are described below.

## CircRNAs and cancers

The studies on the roles of circRNAs in cancer are still in their infancy. The full impact of circRNAs on cancer remains unclear. Herein, we discuss recent advances in circRNA discovery, biological roles, molecular mechanism (Additional file 1: Table S1), and the potential of using circRNAs as cancer biomarkers including the correlation between circRNAs expression and clinical characteristics as well as their diagnostic and predictive values (Additional file 2: Table S2).

## Digestive system cancer

### CircRNAs and esophageal cancer

Using bioinformatic analyses, Li et al. have identified a circRNA containing several exons of the itchy E3 ubiquitin-protein ligase (ITCH), termed cir-ITCH [65], which shares several common miRNA-binding sites with *ITCH* mRNA. ITCH is an important molecule in the Wnt/β-catenin pathway, which regulates protein stability, immune responses, and tumor development. The target genes of ITCH (including p63, p73, Dvl2, and Notch1) are closely associated with tumor formation and chemotherapy sensitivity [66, 67]. Li et al. analyzed the expression of cir-ITCH in 684 esophageal squamous cell carcinoma (ESCC) tissues and adjacent non-cancerous tissues by using real-time quantitative polymerase chain reaction (qPCR) and found that the expression of cir-ITCH was significantly down-regulated in ESCC tissues compared to that in adjacent non-cancerous tissues. Cir-ITCH competitively bound to tumor-associated miRNAs (miR-7, miR-17, and miR-214) to up-regulate the expression of ITCH, promoting the ubiquitination and degradation of phosphorylated Dvl2, and thereby inhibiting the activation of Wnt/β-catenin pathway [65]. Xia et al. found that hsa_circ_0067934 was over-expressed in ESCC tissues, and its high expression was correlated with poor differentiation and advanced stage [68]. In vitro siRNA-silencing of hsa_circ_0067934 could induce cell cycle arrest and inhibit the proliferation and migration of ESCC cells. In addition, in a microarray analysis of circRNA profiles in radiation-sensitive and -resistant human esophageal cancer cell lines, Su et al. showed that the expression of 57 circRNAs was significantly up-regulated whereas that of 17 circRNAs was significantly down-regulated in the radiation-resistant ESCC cells [69]. The results of KEGG analysis showed that over 400 target genes of the differentially expressed circRNAs were enriched in the wnt signaling pathway. These findings suggest that the deregulated expression of circRNAs is closely associated with the development and progression of ESCC.

### CircRNAs and gastric cancer

Li et al. found that the expression of hsa_circ_002059 was significantly down-regulated in gastric cancer tissues [70]. In particular, the expression of hsa_circ_002059 in the plasma of post-operative patients with gastric cancer was lower than that in pre-operative patients. Low expression of hsa_circ_002059 was correlated with distant metastasis and TNM stage. Hsa_circ_002059 stably exists in the plasma of gastric cancer patients, supporting its potential as a biomarker. Chen et al. found that hsa_circ_0000190 was down-regulated in the gastric cancer tissues and the plasma samples of gastric cancer patients and its expression level was associated with tumor size, lymphatic metastasis, distal metastasis, and TNM stage [71]. Chen et al. showed that the expression of circPVT1 was elevated in gastric cancer tissues [72]. CircPVT1 could be used as an independent prognostic marker for the overall survival and disease-free survival time of gastric cancer patients. CircPVT1 promotes cell proliferation by acting as a sponge for miR-125. Hsa_circ_0000096 expression levels were significantly lower in gastric cancer tissues and gastric cancer cell lines. The knockdown of hsa_circ_0000096 reduced the expression of cyclin D1, cyclin-dependent kinase 6 (CDK6), matrix metalloproteinase (MMP)-2, and MMP-9 and significantly inhibited cell proliferation and migration [73]. Another study showed that the expression of two circRNAs (hsa_circRNA_400071 and hsa_circRNA_000792) was up-regulated and the expression of three circRNAs (hsa_circRNA_001959, hsa_circRNA_400066 and hsa_circRNA_001066) was down-regulated in gastric cancer [74]. Hsa_circ_0001895 was found to be down-regulated in gastric cancer tissues, and its expression levels were significantly correlated with tumor differentiation and histological type [75]. Hsa_circ_0014717 was also significantly down-regulated in gastric cancer tissues. Its levels in gastric cancer tissues were related to tumor stage and distal metastasis. More importantly, hsa_circ_0014717 could be detected in gastric juice with high stability [59]. In addition, Zhang et al. demonstrated that a four-circRNA-based classifier could serve as a predictive marker for early recurrence of gastric cancer after radical surgery [76]. These results indicate that the abnormal expression of circRNAs may be novel and non-invasive biomarkers for the diagnosis and prognosis of gastric cancer.

### CircRNAs and colorectal cancer

Bachmayr-Heyda et al. performed RNA sequencing to analyze the differentially expressed circRNAs between colorectal cancer tissues and normal adjacent tissues. They found that the expression of 11 circRNAs was up-regulated whereas that of 28 circRNAs was down-regulated in colorectal cancer tissues [77]. Furthermore, the ratio of some circRNAs to linear RNAs in the cancer tissues (circ0817/CUL5, circ3204/USP3, circ6229/METTL3, and circ7374/TNS4) was lower than that in the normal tissues. Guo et al. identified differentially expressed circRNAs in colorectal cancer by using microarray. In comparison with that in the normal adjacent tissues, the expression of 412 circRNAs in colorectal cancer tissues was up-regulated whereas that of 480 circRNAs was down-regulated [78]. The expression of hsa_circ_0000069 was significantly up-regulated in colorectal cancer tissues and colorectal cancer cell lines. Wang et al. showed that the expression of hsa_circ_001988 was down-regulated in colorectal cancer and was related to tumor differentiation and perineural invasion [79]. Zhang et al. demonstrated that the expression of hsa_circRNA_103809 and hsa_circRNA_104700 was significantly down-regulated in colorectal cancer tissues and their expressions level was closely associated with cancer metastasis [80].

Hsiao et al. demonstrated that circCCDC66 expression was up-regulated in colon cancer [81]. The results of gene function studies showed that circCCDC66 was involved in cell proliferation, migration, and invasion. CircCCDC66 could function as a miRNA sponge to protect *MYC* mRNA from degradation by miRNA-33b and miR-93. Circ-BANP was also found to be over-expressed in colorectal cancer. The knockdown of circ-BANP could significantly attenuate the proliferation of colorectal cancer cells [82]. Hsa_circ_ 001569 could act as a positive regulator of colorectal cancer cell proliferation and invasion. By acting as a sponge of miR-145, hsa_circ_001569 up-regulated the expression of its targets E2F5, BAG4 and FMNL2 [83]. In addition, Huang et al. reported the low expression of cir-ITCH and its role as a microRNA sponge in colorectal cancer. Cir-ITCH could inhibit the expression of c-myc and cyclin D1 [84], which are overexpressed in a variety of tumors including colorectal cancer [85]. Circular antisense RNA, CDR1as, could act as a miRNA sponge to maintain the expression of its host gene *CDR1* [86]. CDR1as contains more than 70 miR-7 MREs and competitively binds to miR-7, which in turn regulates the expression of the miR-7 target genes [87]. CDR1as is therefore also termed as ciRS-7. A recent study suggested that CDR1as was highly expressed in CRC tissues. The expression level of CDR1as is positively associated with tumor size, TNM stage, lymph node metastasis, and poor overall survival (OS) [88]. CDR1as knockdown suppressed colorectal cancer cell proliferation and invasion via inhibiting the activities of miR-7 targets including EGFR and IGF-1R [89]. Li et al. suggested that a large number of circRNAs could be detected in exosomes. The circRNAs found in the serum exosomes showed specificity for colorectal cancer, suggesting that circRNAs in exosomes may be used as biomarkers for colorectal cancer [60]. In summary, these studies indicate that circRNAs are associated with colorectal cancer progression and the differential expression of circRNAs in colorectal cancer tissues, plasma, and serum exosomes provides novel biomarkers for colorectal cancer.

### CircRNAs and liver cancer

Shang et al. performed microarray analyses of circRNA expression in liver cancer tissues and normal adjacent tissues and found that 61 circRNAs were differentially expressed between liver cancer tissues and adjacent normal tissues, among which 26 circRNAs were up-regulated and 35 circRNAs were down-regulated [90]. In particular, hsa_circ_0005075 was up-regulated in liver cancer tissues and showed a high diagnostic value with an AUC of 0.94. In addition, the bioinformatic prediction for circRNA-miRNA interaction networks and gene ontology indicated that hsa_circ_0005075 might be involved in cell adhesion during the development of liver cancer. Qin et al. found that the expression of hsa_circ_0001649 was down-regulated in hepatocellular carcinoma (HCC) tissues [91]. The expression level of hsa_circ_0001649 was correlated with tumor size and tumor emboli in HCC tissues. Yao et al. found that the expression of circZKSCAN1 was significantly lower in HCC samples than that in peritumoral tissues [60]. The expression level of circZKSCAN1 significantly varied in patients with different tumor numbers, cirrhosis, vascular invasion, microscopic vascular invasion, and tumor grade. The over-expression of circZKSCAN1 repressed HCC progression in vitro and in vivo. Fu et al. reported that the expression levels of hsa_circ_0004018 and hsa_circ_0005986 in HCC were significantly lower than that in adjacent noncancerous tissues [61, 62]. The expression levels of hsa_circ_0004018 and hsa_circ_0005986 were correlated with tumor size, differentiation, and TNM stage. Hsa_circ_0003570 was also found down-regulated in HCC tissues, and its expression level was gradually decreased in chronic hepatitis (CH), liver cirrhosis (LC), and HCC [63]. The study by Han et al. showed that circMTO1 was significantly down-regulated in HCC tissues and circMTO1 could suppress HCC progression by acting as the sponge of oncogenic miR-9 to promote p21 expression [92]. Intratumoral administration of circMTO1 siRNA promoted HCC tumor growth in vivo, suggesting that circMTO1 could be a potential target in HCC treatment. The decreased expression of circMTO1 was significantly correlated to poor prognosis in HCC patients, suggesting that circMTO1 may serve as a prognostic biomarker. In addition, Yu et al. found that the expression of CDR1as was up-regulated in liver cancer tissues whereas the expression of miR-7 was down-regulated, suggesting that the expression of CDR1as and miR-7 is negatively correlated [93]. CDR1as interacts with miR-7 to derepress the expression of *CCNE1* and *PIK3CD* genes, thereby promoting the proliferation and invasiveness of liver cancer cells. The study by Xu et al. indicated that the high expression of CDR1as in HCC tissues was significantly correlated to microvascular infiltration (MVI) [94]. These studies indicate that circRNAs may participate in the pathogenesis of liver cancer through multiple mechanisms.

## Urinary system cancer

### CircRNAs and bladder cancer

The results of a circRNA microarray study by Zhong et al. suggested that the expression of 285 circRNAs was up-regulated whereas that of 184 circRNAs was down-regulated in the bladder cancer tissues compared to normal adjacent tissues [95]. qRT-PCR results showed that the expression of circFAM169A (hsa_circ_0007158) and circTRIM24 (hsa_circ_0082582) was down-regulated whereas that of cycTCF25 (hsa_circ_0041103), circZFR (hsa_circ_0072088), circPTK2 (hsa_circ_0005273), and circBC048201 (hsa_circ_0061265) was up-regulated in bladder cancer tissues. CircTCF25 over-expression could down-regulate the activities of miR-103a-3p and miR-107 and increase the expression of CDK6, promoting the proliferation and migration of bladder cancer cells.

### CircRNAs and kidney cancer

Wang et al. identified a new circRNA (termed circHIAT1) that was down-regulated in clear cell renal cell carcinoma (ccRCC) tissues [96]. CircHIAT1 could bind to miR-195-5p/29a-3p/29c-3p to upregulate CDC42 expression. The activation of androgen receptor (AR) suppressed circHIAT1 expression, resulting in decreased CDC42 expression and enhanced ccRCC cell migration and invasion. The AR/circHIAT1/CDC42 signaling pathway may be developed as a new target for the therapy of ccRCC metastasis.

## Head and neck cancer

### CircRNAs and oral cancer

In a comprehensive circRNA microarray analyses for human oral squamous cell carcinoma (OSCC), circRNA_100290 was identified as an up-regulated circRNA in OSCC tissues. The knockdown of circRNA_100290 decreased the expression of CDK6 and inhibited OSCC cell proliferation. CircRNA_100290 could function as a competing endogenous RNA to regulate CDK6 expression through sponging up miR-29b family members [97].

### CircRNAs and hypopharyngeal cancer

The study by Han et al. showed that 2392 circRNAs were differentially expressed in the hypopharyngeal squamous cell carcinoma (HSCC) tissues [98]. Among them, 1304 circRNAs were up-regulated and 1088 circRNAs were down-regulated in HSCC tissues. The expression levels of hsa_circ_0058106, hsa_circ_0058107, and hsa_circ_0024108 were significantly higher in HSCC tissues. Meanwhile, the expression levels of hsa_circ_0036722, hsa_circ_0002260, and hsa_circ_0001189 were significantly decreased in HSCC tissues. The roles of these circRNAs in HSCC have not been well characterized.

### CircRNAs and laryngeal cancer

The study by Xuan et al. showed that 698 circRNAs were differentially expressed in laryngeal squamous cell carcinoma (LSCC) tissues, including 302 up-regulated and 396 down-regulated circRNA transcripts [99]. In particular, hsa_circRNA_100855 was up-regulated in LSCC tissues. The high level of hsa_circRNA_100855 was associated with lymph node metastasis and advanced clinical stage. Conversely, hsa_circRNA_104912 was significantly down-regulated in LSCC tissues. LSCC patients with cervical lymph node metastasis, poor differentiation, or advanced clinical stage showed low level of hsa_circRNA_104912. These studies indicate that circRNAs may play an important role in the development of LSCC and might contribute to the diagnosis and prognosis of this disease.

## Respiratory system cancer

### CircRNAs and lung cancer

Wan et al. found that cir-ITCH was down-regulated in lung cancer tissues and the over-expression of cir-ITCH could inhibit lung cancer cell proliferation [100]. As that observed in colorectal cancer, cir-ITCH also plays a tumor suppressive role by regulating the activities of miR-7 and miR-214, which up-regulates the expression level of ITCH and inhibits the Wnt pathway, leading to reduced lung cancer cell proliferation. Yao et al. found that circRNA_100876 was up-regulated in non-small cell lung cancer (NSCLC) tissues [101]. The high level of circRNA_100876 was correlated to lymph node metastasis and tumor stage in NSCLC. Moreover, the overall survival time for NSCLC patients with high level of circRNA_100876 was significantly shorter than those patients with low level of circRNA_100876.

## Brain cancer

### CircRNAs and glioma

cZNF292 was identified as a circRNA expressed in endothelial cells under hypoxic condition. In vitro inhibition of cZNF292 expression could reduce tube formation and endothelial cell germination [102]. Yang et al. found that cZNF292 was also expressed in glioma cells and that the silencing of cZNF292 expression could inhibit glioma cell proliferation [103]. The knockdown of cZNF292 inhibited Wnt/β-catenin signaling and induced cell cycle arrest. The expression of circ-TTBK2 but not linear TTBK2 was elevated in glioma tissues. Circ-TTBK2 over-expression promoted cell proliferation, migration, and invasion, while inhibited cell apoptosis [104]. Circ-TTBK2 plays an oncogenic role in glioma cells by acting as a miR-217 sponge. In addition, circBRAF was significantly down-regulated in glioma patients with high pathological grade. The high level of circBRAF was an independent biomarker for predicting good progression-free survival and overall survival in glioma patients [105].

## Blood system cancer

### CircRNAs and leukemia

Li et al. identified a large number of circRNAs that were aberrantly expressed in leukemia by using circRNA microarray [106]. Three circRNAs (hsa_circ_0035381, hsa_circ_0004136 and hsa_circ_0058058) were up-regulated and two circRNAs (hsa_circ_0017446 and hsa_circ_0004277) were down-regulated in acute myeloid leukemia patients. The expression level of hsa_circ_0004277 was down-regulated in newly diagnosed AML patients. Additionally, when the patients achieved complete remission (CR), the expression level of hsa_circ_0004277 was increased. However, in relapsed-refractory patients after CR stage, the expression of hsa_circ_0004277 was down-regulated again. These results reveal a dynamic expression of hsa_circ_0004277 during the progression of AML, thus offering a potential biomarker for evaluating the response of AML to therapeutic interventions. In another study, Guarnerio et al. found that fusion circRNAs (f-circRNA), derived from cancer-associated chromosomal translocations, contributed to cellular transformation, promoted cell viability and resistance to therapy, and had tumor-promoting roles in animal models [9].

## CircRNAs and other tumors

The deregulated expression of circRNAs has also been observed in other cancers, such as basal cell carcinoma, pancreatic ductal adenocarcinoma, breast cancer, and ovarian cancer [107–111]. Sand et al. analyzed the expression of circRNAs in basal cell carcinoma and normal adjacent tissues by using microarray and found that 71 circRNAs were differentially expressed in basal cell carcinoma tissues [107]. The expression of 23 circRNAs was significantly up-regulated whereas that of 48 circRNAs was significantly down-regulated in basal cell carcinoma tissues. In addition, 354 MREs were identified in these differentially expressed circRNAs. With the help of RNA sequencing, thousands of circular transcripts were revealed in epithelial ovarian cancer. These circRNAs were enriched for potentially effective miRNA seed matches. Moreover, a significantly larger number of circRNAs than mRNAs are found to be differentially expressed in metastatic tumor tissues compared to primary tumor tissues [111].

## Research strategies for circRNAs

Currently, the studies on circRNA and disease have emerged as a new filed. Various methods have been developed and used to detect circRNA expression and investigate their functions (Fig. 4). The researchers can identify target circRNAs by using RNA sequencing and microarray. The validation methods for circRNA expression mainly include quantitative real-time PCR, droplet digital PCR, northern blotting, and fluorescence in situ hybridization. For functional study, the researchers generally use gene over-expression and knockdown strategies to manipulate circRNA expression. For mechanism study, bioinformatic prediction, luciferase reporter assay, RNA immunoprecipitation, and RNA pull down combined with mass spectrometry are performed to reveal circRNA-miRNA and circRNA-protein interactions. To study the protein-coding potential of a circRNA, the researchers could predict N6-methyladenosin, internal ribozyme entry site (IRES), and open reading frame in circRNA by bioinformatic analyses. Ribosome footprinting, ribosome IP, m6A IP, mass spectrometry, and western blot are generally used for the validation study [50–52]. Several databases have been developed to provide the basic information about circRNAs and their potential regulatory networks [21, 31, 112–118] (Table 2). The researchers can use circBase to download the sequence of a circRNA of interest and clarify its position in the genome and its expression pattern in various tissues and cells. CircInteractome can be used to design primers for circRNA detection and predict the interacting microRNAs and proteins. CircNet can be used to study circRNAs for certain target genes or microRNAs. Circ2Traits is useful for disease-related circRNA studies. With the help of these online databases, the researchers can analyze the differential expression of circRNAs between tissue samples, predict the potential binding sites of miRNAs on the circRNA, and explore the role of circRNA in physiological and pathological processes.

**Table 2:** Online circRNA databases

Name	Description	Website	Reference
circBase	A comprehensive database for public circRNA datasets	http://www.circbase.org	[112]
CircInteractome	proteins or miRNAs, as well as primer design and siRNA design	http://circinteractome.nia.nih.gov	[113]
Circnet	A database of circular RNAs derived from transcriptome sequencing data	http://circnet.mbc.nctu.edu.tw/	[114]
circ2Traits	A knowledgebase of human circRNAs associated with diseases or traits	http://gyanxet-beta.com/circdb/	[115]
deepBase v2.0	A database of small RNAs, long non-coding RNAs and circular RNAs from deep sequencing	http://rna.sysu.edu.cn/deepBase/	[116]
starBase v2.0	circRNA database for miRNA-circRNA interactions	http://starbase.sysu.edu.cn/	[117]
CIRCpedia	Annotating alternative back-splicing and alternative splicing in circRNAs	http://www.picb.ac.cn/rnomics/circpedia	[21]
circRNADb	A comprehensive database for human circular RNAs with protein-coding annotations.	http://reprod.njmu.edu.cn/circrnadb	[118]
TSCD	A database of tissue-specific circular RNAs in the human and mouse genomes	http://gb.whu.edu.cn/TSCD	[31]

## Conclusions

In this review, we briefly summarized the formation, characteristics, biological functions and clinical values of circRNAs with an emphasis on cancer. CircRNAs exhibit a high degree of tissue and cell specificity, and are closely related to certain physiological and pathological conditions, indicating that the formation of circRNAs is not an accidental and random event but a strictly controlled biological process. Although the formation models of circRNAs have been preliminarily proposed, extensive efforts are required to fully understand the mechanism responsible for the production of circRNAs, including the biogenesis of nascent circRNAs, the secondary structures of circRNAs, and the relationship between different RNA products of the same host gene.

The current studies mainly focus on the unique expression pattern of circRNAs in cancer and the biological roles of circRNAs in cancer development and progression. CircRNAs can regulate gene expression at transcriptional and post-transcriptional levels. Some circRNAs even can translate proteins. CircRNAs can function as miRNA sponge, which is found to be a mechanism for its role in cancer. The role of circRNAs in regulating miRNAs makes the ceRNA network more complete and complicated. However, since most circRNAs are present in low abundance and are of short lengths [33], ceRNA may not represent the main role of circRNAs. In the future, the other mechanisms responsible for the functions of circRNAs in cancer, such as the regulation of gene or protein activities, need to be further explored. In addition, circular-to-linear RNA expression is generally higher in the blood compared to tissues, suggesting that the cells may secrete circRNAs via exosomes into the blood [58]. The circulating circRNAs may have important roles in the cellular communication. The functional roles of exosomal circRNAs warrant further investigation.

The detection of circRNA in cancer mainly focus on tissue samples. More easily acquired and non-invasive clinical samples (blood, urine, saliva, etc.) and samples closely related to the disease (gastric juice, cerebrospinal fluid, and synovial fluid) should be tested for circRNA expression in the future research. The sample processing, detection method uniformity, and cut-off value determination need to be optimized for developing circRNAs as clinical diagnosis biomarkers. Combined detection may also be considered to achieve better diagnostic results (including the combined detection of different circRNAs and the combined detection of circRNAs and traditional diagnostic markers). In addition, circRNAs are also considered as potential targets for cancer therapy. Considering the potent roles of circRNAs in cancer, targeting circRNAs may help improve the efficacy of cancer therapy.

## Additional files

Additional file 1: Table S1. The expression and function of circRNAs in cancer. (DOCX 25 kb)

Additional file 2: Table S2. The potential of circRNAs as cancer biomarkers. (DOCX 32 kb)

## Abbreviations

ADAR1: Adenosine deaminase acting on RNA-1
ANRIL: Antisense non-coding RNA in the INK4 locus
ccRCC: Clear cell renal cell carcinoma
ceRNAs: Competing endogenous RNAs
circRNAs: Circular RNAs
DHX9: DExH-box helicase 9
ESCC: Esophageal squamous cell carcinoma
f-circRNA: Fusion circRNAs
FUS: Fused in sarcoma
HCC: Hepatocellular carcinoma
HDAg: Hepatitis D virus antigen
IRES: Internal ribosome entry site
ITCH: Itchy E3 ubiquitin-protein ligase
LSCC: Laryngeal squamous cell carcinoma
*MBL*: Muscleblind gene
MRE: microRNA response element
PcG: Polycomb group
qPCR: Real-time quantitative polymerase chain reaction
RBPs: RNA-binding proteins
snRNPs: Small nuclear ribonucleoproteins
