# Supplementary material for: Circular RNAs: emerging cancer biomarkers and targets
Source: J Exp Clin Cancer Res. 2017 Nov 2;36:152. doi: 10.1186/s13046-017-0624-z (PMC5667461; doi:10.1186/s13046-017-0624-z)
Supplement: Supplementary file 1 — The expression and function of circRNAs in cancer. (DOCX 25 kb) [file 13046_2017_624_MOESM1_ESM.docx]

[**Table S1**](https://static-content.springer.com/esm/art%3A10.1186%2Fs13046-017-0539-8/MediaObjects/13046_2017_539_MOESM1_ESM.tif)**. The expression and function of circRNAs in cancer.**

| Cancer type | CircRNA | Regulation | Function | Mechanism | MicroRNA binding site (n) | Reference |
| --- | --- | --- | --- | --- | --- | --- |
| **Digestive system cancer** |  |  |  |  |  |  |
| Esophageal cancer | hsa_circ_0001141 *(cir-ITCH)* | down | Cell proliferation | cir-ITCH- miR-7/miR-17/miR-214- ITCH- Wnt/β-catenin |  | [65] |
|  | has_circ_0067934 | up | Cell proliferation and migration | - |  | [68] |
|  |  |  |  |  |  |  |
| Gastric cancer | hsa_circ_0000140 *(hsa_circ_002059)* | down | - | - |  | [70] |
|  | hsa_circ_0000190 | down | - | - |  | [71] |
|  | circPVT1 | up | Cell proliferation | circPVT1-miR-125 | 2 | [72] |
|  | hsa_circ_0000096 | down | Cell proliferation and migration | cyclin D1, CDK6, MMP-2 and MMP-9 |  | [73] |
|  | hsa_circ_0092283 *(hsa_circ_400071)* | up | - | - |  | [74] |
|  | hsa_circ_0000291 *(hsa_circ_000792)* | up | - | - |  | [74] |
|  | hsa_circ_0001539 *(hsa_circ_001959)* | down | - | - |  | [74] |
|  | hsa_circ_0092330 *(hsa_circ_400066)* | down | - | - |  | [74] |
|  | hsa_circ_0000627 *(hsa_circ_001066)* | down | - | - |  | [74] |
|  | hsa_circ_0001895 | down | - | - |  | [75] |
|  | hsa_circ_0014717 | down | - | - |  | [59] |
|  |  |  |  |  |  |  |
| Colorectal cancer | hsa_circ_0000069 | up | - | - |  | [78] |
|  | hsa_circ_0001988 *(hsa_circ_001988)* | down | - | - |  | [79] |
|  | hsa_circ_0072088 *(hsa_circ_103809)* | down | - | - |  | [80] |
|  | hsa_circ_0005273 (hsa_circ_104700) | down | - | - |  | [80] |
|  | CCDC66 | up | Cell proliferation, migration, and invasion | circCCDC66-miR-33b/miR-93-myc |  | [81] |
|  | [Circular BANP](javascript:;) | up | - | - |  | [82] |
|  | hsa_circ_0001569 (hsa_circ_001569) | up | Cell proliferation and invasion | CircRNA-001569-miR145-E2F5/BAG4/ FMNL2 | 1 | [83] |
|  | hsa_circ_0001141 *(cir-ITCH)* | down | Cell proliferation | cir-ITCH-miR-7 / miR-20a-ITCH- Wnt/β-catenin |  | [84] |
|  | [hsa_circ_0001946](http://www.circbase.org/cgi-bin/singlerecord.cgi?id=hsa_circ_0001946) (CDR1as/ciRS-7) | up | - | - | 73 | [86] |
|  | circ-KLDHC10 | up | - | - |  | [60] |
|  |  |  |  |  |  |  |
| Liver cancer | hsa_circ_0005075 | up | - | - |  | [90] |
|  | hsa_circ_0001649 | down | - | - |  | [91] |
|  | circZKSCAN1 | down | Cell growth, migration, and invasion | - |  | [61] |
|  | hsa_circ_0004018 | down | - | - |  | [62] |
|  | hsa_circ_ 0005986 | down | Cell proliferation | hsa_circ_0005986-miR-129-5p-Notch1 | 1 | [63] |
|  | hsa_circ_0003570 | down | - | - |  | [64] |
|  | circMTO1 | down | Cell proliferation and invasion | circMTO1-miR-9-p21 |  | [92] |
|  | [hsa_circ_0001946](http://www.circbase.org/cgi-bin/singlerecord.cgi?id=hsa_circ_0001946) (CDR1as/ciRS-7) | up | Cell proliferation and invasion | CDR1as-miR-7-PIK3CD/CUL5 |  | [93] |
|  |  |  |  |  |  |  |
| **Urinary system cancer** |  |  |  |  |  |  |
| Bladder cancer | hsa_circ_0072088 | up | - | - |  | [95] |
|  | hsa_circ_0005273 | up | - | - |  | [95] |
|  | hsa_circ_0061265 | up | - | - |  | [95] |
|  | hsa_circ_0041103 | up | Cell proliferation and migration | circTCF25-miR-  103a-3p/miR-107-CDK6 | 1/1 | [95] |
|  | hsa_circ_0007158 | down | - | - |  | [95] |
|  | hsa_circ_0082582 | down | - | - |  | [95] |
|  |  |  |  |  |  |  |
| Clear cell renal cell carcinoma | [hsa_circ_0000096](http://www.circbase.org/cgi-bin/singlerecord.cgi?id=hsa_circ_0000096) (circHIAT1) | down | - | circHIAT1-miR-195-5p/ miR-29a-3p/ miR-29c-3p-CDC42 | 2/1/1 | [96] |
|  |  |  |  |  |  |  |
| **Head and neck cancer** |  |  |  |  |  |  |
| Oral cancer | hsa_circRNA_100290 | up | Cell proliferation | circRNA100290-miR-29b- CDK6 | 2 | [97] |
| Hypopharyngeal cancer | hsa_circ_0058106 | up | - | - |  | [98] |
|  | hsa_circ_0058107 | up | - | - |  | [98] |
|  | hsa_circ_0024108 | up | - | - |  | [98] |
|  | hsa_circ_0036722 | down | - | - |  | [98] |
|  | hsa_circ_0001189 | down | - | - |  | [98] |
|  | hsa_circ_0002260 | down | - | - |  | [98] |
|  |  |  |  |  |  |  |
| Laryngeal cancer | hsa_circ_0023033 *(hsa_circ_100855)* | up | - | - |  | [99] |
|  | hsa_circ_0088475 *(hsa_circ_104912)* | down | - | - |  | [99] |
| **Respiratory system cancer** |  |  |  |  |  |  |
| Lung cancer | hsa_circ_0001141 *(cir-ITCH)* | down | - | cir-ITCH-miR-7/ miR-214-ITCH- Wnt/𝛽-catenin | 73 | [100] |
|  | hsa_circ_0023249 *(hsa_circ_100876)* | up | - | - |  | [101] |
|  |  |  |  |  |  |  |
| **Brain cancer** |  |  |  |  |  |  |
| Glioma | cZNF292 |  | Cell proliferation | cZNF292-Wnt/β-catenin |  | [103] |
|  | circ-TTBK2 | up | Cell growth, migration, and apoptosis | circ-TTBK2-miR217-HNF1β/Derlin-1 | 1 | [104] |
|  | circBRAF | down | - | - |  | [105] |
|  |  |  |  |  |  |  |
| **Blood system cancer** |  |  |  |  |  |  |
| Acute myeloid leukemia | hsa_circ_0004277 | down | - | - |  | [106] |
|  | f-circPR | - | Cell proliferation | - |  | [9] |
|  | f-circM9 | - | Cell proliferation | - |  | [9] |
