# Supplementary material for: Circular RNAs: emerging cancer biomarkers and targets
Source: J Exp Clin Cancer Res. 2017 Nov 2;36:152. doi: 10.1186/s13046-017-0624-z (PMC5667461; doi:10.1186/s13046-017-0624-z)
Supplement: Supplementary file 2 — The potential of circRNAs as cancer biomarkers. (DOCX 32 kb) [file 13046_2017_624_MOESM2_ESM.docx]

[**Table S2**](https://static-content.springer.com/esm/art%3A10.1186%2Fs13046-017-0539-8/MediaObjects/13046_2017_539_MOESM1_ESM.tif)**. The** **potential of circRNAs as cancer biomarkers.**

| **Cancer type** | **CircRNA** | **Sample type** | **Number** | **Regulation** | **AUC** | **Sensitivity** | **Specificity** | **Clinical characteristics** | **Reference** |
| --- | --- | --- | --- | --- | --- | --- | --- | --- | --- |
| **Digestive system cancer** |  |  |  |  |  |  |  |  |  |
| Esophageal cancer | hsa_circ_0001141 *(cir-ITCH)* | Tissue  (tumor vs. non-tumor) | 684 | down |  |  |  |  | [65] |
|  | hsa_circ_0067934 | Tissue  (tumor vs. non-tumor) | 51 | up |  |  |  | Tumor differentiation and TNM stage | [68] |
| Gastric cancer | has_circ_0000140 *(hsa_circ_002059)* | Tissue  (tumor vs. non-tumor) | 101 | down | 0.73 | 0.81 | 0.62 | Age, Gender, and TNM stage | [70] |
|  |  | Plasma  (Preoperative vs. postoperative) | 36 | down |  |  |  |  |  |
|  | hsa_circ_0000190 | Tissue  (tumor vs. non-tumor) | 104 | down | 0.75 | 0.72 | 0.68 | Tumor size, TNM stage, and  CA19-9 level. | [71] |
|  |  | Plasma  (tumor vs. healthy control) | 104 | down | 0.60 | 0.41 | 0.88 | CEA |  |
|  |  | combination  (plasma and tissue) |  |  | 0.78 | 0.71 | 0.75 |  |  |
|  | circPVT1 | Tissue  (tumor vs. non-tumor) | 187 | up |  |  |  | Prognosis | [72] |
|  | hsa_circ_0000096 | Tissue  (tumor vs. non-tumor) | 101 | down | 0.82 |  |  |  | [73] |
|  |  | Combination  (hsa_circ_0000096 and has_circ_0000140) |  |  | 0.91 |  |  |  |  |
|  | hsa_circ_0092283 *(hsa_circ_400071)* | Tissue  (tumor vs. non-tumor) | 8 | up |  |  |  |  | [74] |
|  | hsa_circ_0000291 *(hsa_circ_000792)* | Tissue  (tumor vs. non-tumor) | 8 | up |  |  |  |  | [74] |
|  | hsa_circ_0001539 *(hsa_circ_001959)* | Tissue  (tumor vs. non-tumor) | 8 | down |  |  |  |  | [74] |
|  | hsa_circ_0092330 *(hsa_circ_400066)* | Tissue  (tumor vs. non-tumor) | 8 | down |  |  |  |  | [74] |
|  | hsa_circ_0000627 *(hsa_circ_001066)* | Tissue  (tumor vs. non-tumor) | 8 | down |  |  |  |  | [74] |
|  | hsa_circ_0001895 | Tissue  (tumor vs. non-tumor) | 96 | down | 0.792 | 67.8% | 85.7% | Borrmann type, CEA (tissue), Tumor differentiation | [75] |
|  |  | Tissue  (GC vs. GD vs. healthy control) | 96/30/35 | down |  |  |  |  |  |
|  | hsa_circ_0014717 | Tissue  (tumor vs. non-tumor) | 96 | down | 0.696 | 59.38% | 59.38% | TNM stage, CEA (tissue), and CA19-9 (tissue) | [59] |
|  |  | Gastric Juices  (GC vs. CAG vs. GU vs. healthy control) | 39/15/30/38 | down |  |  |  |  |  |
| Colorectal cancer | hsa_circ_0000069 | Tissue  (tumor vs. non-tumor) | 30 | up |  |  |  | Age and TNM stage | [78] |
|  | hsa_circ_0001988 *(hsa_circ_001988)* | Tissue  (tumor vs. non-tumor) | 31 | down | 0.788 | 0.68 | 0.73 | Tumor differentiation and  Perineural invasion | [79] |
|  | hsa_circ_0072088 *(hsa_circ_103809)* | Tissue  (tumor vs. non-tumor) | 170 | down | 0.699 |  |  | TNM stage | [80] |
|  | hsa_circ_0005273 *(hsa_circ_104700)* | Tissue  (tumor vs. non-tumor) | 170 | down | 0.616 |  |  | TNM stage | [80] |
|  | CCDC66 | Tissue  (tumor vs. non-tumor) | 48 | up | 0.884 |  |  | Prognosis | [81] |
|  | [Circular BANP](javascript:;) | Tissue  (tumor vs. non-tumor) | 35 | up |  |  |  |  | [82] |
|  | hsa_circ_0001569 *(hsa_circ_001569)* | Tissue  (tumor vs. non-tumor) | 30 | up |  |  |  | Tumor differentiation and TNM stage | [83] |
|  | hsa_circ_0001141 *(cir-ITCH)* | Tissue  (tumor vs. non-tumor) | 45 | down |  |  |  |  | [84] |
|  | [hsa_circ_0001946](http://www.circbase.org/cgi-bin/singlerecord.cgi?id=hsa_circ_0001946) *(CDR1as/ciRS-7)* | Tissue  (tumor vs. non-tumor) | 40 | up |  |  |  | TNM stage and Prognosis | [86] |
|  | circ-KLDHC10 | Serum  (CC vs. healthy control) | 11/3 | up |  |  |  |  | [60] |
| Liver cancer | hsa_circ_0005075 | Tissue  (tumor vs. non-tumor) | 60 | up |  |  |  | Tumor size | [90] |
|  | hsa_circ_0001649 | Tissue  (tumor vs. non-tumor) | 89 | down | 0.63 | 0.81 | 0.69 | Tumor size and Tumor embolus | [91] |
|  | circZKSCAN1 | Tissue  (tumor vs. non-tumor) | 102 | down |  |  |  | Tumor numbers, Cirrhosis, Vascular  invasion, and TNM stage | [61] |
|  | hsa_circ_0004018 | Tissue  (tumor vs. non-tumor)  Tissue  (HCC vs. LC vs. CH) | 102  102/63/66 | down  down | 0.848 | 0.716 | 0.815 | AFP (serum), Tumor size, Tumor differentiation, BCLC stage, and TNM stage. | [62] |
|  | hsa_circ_ 0005986 | Tissue  (tumor vs. non-tumor) | 81 | down |  |  |  | Tumor size, Microvascular invasion, and BCLC stage | [63] |
|  | hsa_circ_0003570 | Tissue  (tumor vs. non-tumor)  Tissue  (HCC vs. LC vs. CH) | 107  107/66/71 | down  down |  |  |  | Tumor size,  Tumor differentiation, Microvascular invasion, BCLC stages, TNM stages, and AFP (serum). | [64] |
|  | hsa_circ_0007874 *(circMTO1)* | Tissue  (tumor vs. non-tumor) | 289 | down |  |  |  | Prognosis | [92] |
|  | [hsa_circ_0001946](http://www.circbase.org/cgi-bin/singlerecord.cgi?id=hsa_circ_0001946) *(CDR1as/ciRS-7)* | Tissue  (tumor vs. non-tumor) | 35 | up |  |  |  |  | [93] |
| **Urinary system cancer** |  |  |  |  |  |  |  |  |  |
| Bladder cancer | hsa_circ_0072088 | Tissue  (tumor vs. non-tumor) | 40 | up |  |  |  |  | [95] |
|  | hsa_circ_0005273 | Tissue  (tumor vs. non-tumor) | 40 | up |  |  |  |  | [95] |
|  | hsa_circ_0061265 | Tissue  (tumor vs. non-tumor) | 40 | up |  |  |  |  | [95] |
|  | hsa_circ_0041103 | Tissue  (tumor vs. non-tumor) | 40 | up |  |  |  |  | [95] |
|  | hsa_circ_0007158 | Tissue  (tumor vs. non-tumor) | 40 | down |  |  |  |  | [95] |
|  | hsa_circ_0082582 | Tissue  (tumor vs. non-tumor) | 40 | down |  |  |  |  | [95] |
|  |  |  |  |  |  |  |  |  |  |
| Clear cell renal cell carcinoma | [hsa_circ_0000096](http://www.circbase.org/cgi-bin/singlerecord.cgi?id=hsa_circ_0000096) *(circHIAT1)* | Tissue  (tumor vs. non-tumor) | 40 | down |  |  |  |  | [96] |
| **Head and neck cancer** |  |  |  |  |  |  |  |  |  |
| Oral cancer | hsa_circ_0013339 *(hsa_circ_100290)* | Tissue  (tumor vs. non-tumor) | 5 | up |  |  |  |  | [97] |
| Hypopharyngeal cancer | hsa_circ_0058106 | Tissue  (tumor vs. non-tumor) | 32 | up |  |  |  |  | [98] |
|  | hsa_circ_0058107 | Tissue  (tumor vs. non-tumor) | 32 | up |  |  |  |  | [98] |
|  | hsa_circ_0024108 | Tissue  (tumor vs. non-tumor) | 32 | up |  |  |  |  | [98] |
|  | hsa_circ_0036722 | Tissue  (tumor vs. non-tumor) | 32 | down |  |  |  |  | [98] |
|  | hsa_circ_0001189 | Tissue  (tumor vs. non-tumor) | 32 | down |  |  |  |  | [98] |
|  | hsa_circ_0002260 | Tissue  (tumor vs. non-tumor) | 32 | down |  |  |  |  | [98] |
| Laryngeal cancer | hsa_circ_0023033 *(hsa_circ_100855)* | Tissue  (tumor vs. non-tumor) | 52 | up |  |  |  | TNM stage | [99] |
|  | hsa_circ_0088475 *(hsa_circ_104912)* | Tissue  (tumor vs. non-tumor) | 52 | down |  |  |  | TNM stage and Tumor differentiation | [99] |
| **Respiratory system cancer** |  |  |  |  |  |  |  |  |  |
| Lung cancer | hsa_circ_0001141 *(cir-ITCH)* | Tissue  (tumor vs. non-tumor) | 78 | down |  |  |  | Age | [100] |
|  | hsa_circ_0023249  *(hsa_circ_100876)* | Tissue  (tumor vs. non-tumor) | 101 | up |  |  |  | TNM stage and Prognosis | [101] |
| **Brain cancer** |  |  |  |  |  |  |  |  |  |
| Glioma | cZNF292 |  |  |  |  |  |  |  | [103] |
|  | circ-TTBK2 | Tissue  (tumor vs. non-tumor) | 76/11 | up |  |  |  | Pathological grade | [104] |
|  | circBRAF | Tissue  (tumor vs. non-tumor) | 5 | down |  |  |  | Pathological grade  and Prognosis | [105] |
| **Blood system cancer** |  |  |  |  |  |  |  |  |  |
| Acute myeloid leukemia | hsa_circ_0004277 | Mononuclear cells (BM)  (AML vs. healthy control) | 113/12 | down | 0.95 |  |  | Progressive stage | [106] |
|  | f-circPR |  |  | - |  |  |  |  | [9] |
|  | f-circM9 |  |  | - |  |  |  |  | [9] |

### TNM: tumor-node-metastasis; GC: gastric cancer; GD: gastric dysplasia; GU: gastric ulcer; CC: colorectal cancer; CAG: chronic atrophic gastritis; BCLC: Barcelona Clinic Liver Cancer; AFP: alpha fetoprotein; CH: chronic hepatitis; LC: liver cirrhosis; HCC: hepatocarcinoma; BM: bone marrow; AML: acute myeloid leukemia.
